# From Physical Aggression to Verbal Behavior: Language Evolution and Self-Domestication Feedback Loop

**DOI:** 10.3389/fpsyg.2019.02807

**Published:** 2019-12-18

**Authors:** Ljiljana Progovac, Antonio Benítez-Burraco

**Affiliations:** ^1^Linguistics Program, Department of English, Wayne State University, Detroit, MI, United States; ^2^Department of Spanish Language, Linguistics and Literary Theory (Linguistics), Faculty of Philology, University of Seville, Seville, Spain

**Keywords:** language evolution, self-domestication, reactive/proactive aggression, verbal aggression, neurolinguistics, language disorders, child development, sexual selection

## Abstract

We propose that human self-domestication favored the emergence of a less aggressive phenotype in our species, more precisely phenotype prone to replace (reactive) physical aggression with verbal aggression. In turn, the (gradual) transition to verbal aggression and to more sophisticated forms of verbal behavior favored self-domestication, with the two processes engaged in a mutually reinforcing feedback loop, considering that verbal behavior entails not only less violence and better survival but also more opportunities to interact longer and socialize with more conspecifics, ultimately enabling the emergence of more complex forms of language. Whereas in the case of self-domestication, sexual selection has been proposed to work against physical aggression traits, in the case of verbal insult, the selection has been proposed to work in favor of verbal aggression. The tension between these two seemingly opposing forces gets resolved/alleviated by a tendency to replace physical aggression with verbal aggression and with verbal behavior more generally. This also helps solve the paradox of the Self-Domestication Hypothesis regarding aggression, more precisely why aggression in humans has been reduced only when it comes to reactive aggression, but not when it comes to proactive aggression, the latter exhibiting an increase in the advent of modern language. We postulate that this feedback loop was particularly important during the time period arguably between 200 and 50 kya, when humans were not fully modern, neither in terms of their skull/brain morphology and their behavior/culture nor in terms of their self-domestication. The novelty of our approach lies in (1) giving an active role to early forms of language in interacting with self-domestication processes; (2) providing specific linguistic details and functions of this early stage of grammar (including insult and humor); (3) supplying neurobiological, ontogenetic, and clinical evidence of a link between (reactive) aggression and (reactive) verbal behavior; (4) identifying proxies of the earlier stages in evolution among cognitive disorders; and (5) identifying specific points of contact and mutual reinforcement between these two processes (self-domestication and early language evolution), including reduction in physical aggression and stress/tension, as well as sexual selection.

## Introduction

Here we propose that human self-domestication (the presence in humans of morphological, physiological, behavioral, and cognitive features commonly found in domestic animals) co-evolved with a gradual transition from in-group (reactive) physical aggression to inter-group (complex) verbal behavior *via* (reactive) verbal aggression, in a mutually reinforcing fashion. We explore here in detail the possibility that the emergence of the simplest forms of language/grammar accelerated processes of self-domestication and brain evolution already underway, which in turn fueled the transition to more complex languages. Early verbal creations would have afforded an adaptive (non-violent) way to compete for status and sex (e.g., Progovac and Locke, 2009), accelerating/reinforcing self-domestication, while enhanced self-domestication provided a richer niche for extended communication and language learning enabling the transition to more complex forms of language.

Language – communication relying on syntax and grammar – is usually construed as a human-specific cognitive faculty that resulted from biological changes (e.g., Bolhuis et al., 2014; Chomsky, 2017). As a consequence, its history is generally reconstructed by looking for proxies of language in extinct hominin species and for (deep) homologs of language in extant species. By contrast, emergence and divergence of modern languages across the globe are presumed influenced by the physical environment, and social and cultural practices, with such influences largely confined to non-grammatical, lexical components. As a consequence, the history of languages is traced with a minimal reference to changes in brain, behavior, and cognition.

There is ample evidence that socio-cultural factors do indeed influence the divergence of modern languages, and this goes well beyond the attested effect of social factors on linguistic diversity within a language (as studied by Sociolinguistics) or on the lexicons of world languages (as studied by Anthropological Linguistics). For instance, the number of speakers seemingly contributes to explain the morphological complexity of languages (Lupyan and Dale, 2010). Likewise, computational modeling, experimental work with human learners, and language emergence in certain cultural contexts (like the *homesigns* developed by isolated deaf communities) have shown that core properties of language, such as duality of pattern or compositionality, can emerge by iterated learning and cultural transmission (Sandler et al., 2005; Tamariz and Kirby, 2016) and that the same cognitive and biological biases can result in different language features in different cultural environments (Thompson et al., 2016). Increasingly, however, evidence suggests that language structure also impacts on basic cognitive abilities, such as effects of word order on working memory (Amici et al., 2019). As a consequence, language features, language learning, and cognitive architecture comprise a reinforcing feedback loop (Deacon, 2003; Clarke and Heyes, 2017), wherein genetic changes occurred to accommodate language-specific cognition (Jablonka et al., 2012). The greater cognitive cost of language processing and learning incurred by certain recently evolved languages might have necessitated cognitive adaptation because of the enhanced demands on working memory and executive control (Benítez-Burraco and Kempe, 2018). In brief, we should expect not only that our cognitive architecture accounts for many aspects of the languages we speak, but also that certain language features, resulting from cultural and environmental factors, affect, more or less permanently, our cognitive architecture. These two aspects cannot be detached one from the other.

We have a good understanding of the morphological changes that apparently afforded language readiness, including brain rewiring associated with the globularization of the human skull/brain, which is a distinctive feature of our species when compared to the elongated shape found in Neanderthals and Denisovans (for details, see Boeckx and Benítez-Burraco, 2014a). Likewise, we also appreciate the changes in human behavior and culture that affect language structure and divergence^1^. However, we lack good hypotheses about the feedback loop between these two processes. One possibility is that the biological changes that brought about our species also favored the creation of the niche that enabled the emergence of aspects of language complexity *via* cultural evolution, which in turn affected our biology. Another possibility, not mutually exclusive, is that certain cultural practices affected our biology and paved the way toward specific cognitive changes that enabled the emergence of language complexity. Human self-domestication might have contributed to both processes, the evolution of our language-ready brain, mostly *via* biological mechanisms, and the creation of modern languages mostly *via* cultural mechanisms. Prior proposals linking language evolution with self-domestication in humans (e.g., Thomas and Kirby, 2018) seem to assume a unidirectional causal relationship, whereby self-domestication contributed to the emergence of language readiness and of complex languages. Such proposals have not advanced explicit hypotheses regarding how some specific language expressions/structures would have contributed to self-domestication processes and thus to the biological aspects of human evolution. Here we explore such a possibility in detail.

## The Language Evolution/Self-Domestication Feedback Loop: A Hypothesis

Compared to our primate relatives (perhaps with the exception of bonobos), and to species of extinct hominins, present-day humans exhibit reduced aggression (Herrmann et al., 2011). Morphological changes indicative of reduced aggression appear in the fossil record alongside an increase in cultural artifacts, from around 80,000 years ago (Hare et al., 2012). The human self-domestication hypothesis (Hare, 2017) proposes that these changes evolved when natural selection favored increased in-group prosociality over aggression in human evolution. Accordingly, as a by-product of this selection, present-day humans are thought to exhibit most of the physical, physiological, and behavioral traits commonly found in domesticated strains of animals compared to their wild conspecifics, including reduced cranial robusticity and brain size, neotenic features (mostly affecting the face), reduced sexual dimorphism, reduced aggression, increased playing behavior, enhanced socialization, and reduced responsiveness to stress as measured by cortisol levels (Shea, 1989; Leach, 2003; Somel et al., 2009; Zollikofer and Ponce de León, 2010; Herrmann et al., 2011; Plavcan, 2012; Márquez et al., 2014; Fukase et al., 2015; Stringer, 2016). This is seemingly due to the fact that selection against aggression inhibits the proliferation of the neural crest cells (NCCs), ultimately affecting the development of many body components (Wilkins et al., 2014, but see Sánchez-Villagra and van Schaik, 2019 for some cautionary notes). Less aggressive behavior resulting from our self-domestication might have specifically enhanced learning and teaching opportunities and our capacity for knowledge exchange and group collaboration, ultimately supporting an increase in language complexity *via* a cultural process (Benítez-Burraco and Kempe, 2018 and Thomas and Kirby, 2018).

However, this broad picture has to be properly qualified. In spite of the trend toward increased in-group tolerance and prosociality, demographic pressures during the last part of our history seemingly increased inter-group aggression (Choi and Bowles, 2007). As a consequence, although reactive physical aggression (that which arises from fear or anger) has declined over time, inter-group proactive aggression (which strategically aims to achieve specific outcomes) has increased (Wrangham, 2018). Our proposal, which gives the emergence of language an active role, helps explain this otherwise surprising discrepancy between in-group and inter-group violence, which cannot be explained solely by self-domestication^2^. Interestingly, while proactive aggression seems to be tied to complex language/cognition, derogatory language, like swearing, is typically reactive, reinforcing our idea that it serves well to replace reactive physical aggression, specifically, and that it represents an early stage in the evolution of language complexity under the self-domestication hypothesis^3^.

While some reactive physical aggression persists, it has been largely replaced by reactive verbal aggression. Verbal rituals have persisted throughout recorded history (Locke and Bogin, 2006; Locke, 2009). Such duels with words, as opposed to fists, provide an adaptive way to discharge aggressive dispositions (Marsh, 1978) and to compete without risking physical harm (Locke, 2008). Although verbal duels may be a cathartic purging of aggressive impulses, their beauty, creativity, artistic value, and cultural specificity have also been observed by many (Darmesteter, 1934; Samarin, 1969; and Pagliai, 2009). While linguists tend to focus on the language function of conveying information (and have tended to “sanitize” the language they study, excluding swearing, Bergen, 2016, p. 3), there are other, expressive, esthetic, and profane aspects of language, which are just as relevant in the context of language evolution (Haiman, 2013). Both verbal aggression and creativity are directly relevant to our proposal, showing the multiple adaptive advantages of using linguistic aggression over physical fighting (see section “Emergence of Proto-Syntax and Verbal Aggression (Insult)” for further discussion).

Direct verbal confrontation often makes use of simple forms of language, as illustrated with, e.g., crude compounds consisting of just one verb and one noun [e.g., English *kill-joy*, *pick-pocket, scatter-brain*, *turn-coat, cry-baby*; Serbian *cepi-dlaka* “split-hair,” *vrti-guz* “spin-butt” (fidget), *ispi-čutura* “drink-flask” (drunkard), *jebi-vetar* “screw-wind” (charlatan)]. As such, very simple grammars can suffice for verbal aggression and insult. Significantly, these compounds, which afford a particularly creative strategy for coining names with derogatory reference, have been analyzed as approximations of the earliest stages of grammar, showing both crude syntax and primitive vocabulary (e.g., Progovac and Locke, 2009; Progovac, 2015, 2016). Our hypothesis is that looking at the (gradual) emergence of verbal means of aggression (approximated by this kind of compound) might help illuminate the initial steps of the language evolution/self-domestication feedback loop. These verbal items would have afforded an adaptive (non-violent) way to compete for status and sex, first by derogating existing rivals and placing prospective rivals on notice; and second by demonstrating verbal skills and quick wittedness, both directly relevant for sexual selection (Progovac and Locke, 2009, p. 346)^4^. As a consequence, they would have accelerated/reinforced the effects of self-domestication on human behavior and cognition, promoting the transition to more complex forms of language. These types of verbal forms promise to make just a bit narrower the otherwise enormous chasm separating, on the one hand, expressions of emotion/aggression in animals, and, on the other hand, refined human language, with embedded sentences, and thousands of words expressing various subtleties of meaning. Code (2005, and references therein) offers evidence that swearwords are neurally distinct from the other words, relying both on brain areas where compositional language is processed, and on brain areas which support laughing and crying. In that sense, swearwords straddle the boundary between (animal) calls, which share many properties with laughing and crying, on the one hand, and compositional language, on the other. This reinforces the view that swearwords, which also often feature in insults, are primarily reactive, as are laughter and crying. Given that domestication processes can be long and protracted and not guaranteed to succeed either^5^, it is important that we can identify factors that can reinforce it. According to our view, one of these factors was the gradual emergence of language itself (see also Sánchez-Villagra and van Schaik, 2019 for the importance of considering additional, synergistic factors, including language, in the considerations of self-domestication).

For concreteness, we postulate that this feedback loop was particularly important during the time period roughly between 200 and 50 kya^6^. This is a long time period when humans were not fully modern, neither in terms of their skull/brain morphology (and presumably, their cognitive abilities) and their behavior/culture nor in terms of their self-domestication (see Hare, 2017). During this time period, we propose to correlate the advances in human self-domestication processes with the emergence of simple forms of language/syntax, which were particularly suitable for the expression of verbal aggression. The novelty of our approach lies in (1) giving an active role to early forms of language in interacting with self-domestication processes; (2) providing specific details and functions of this early stage of grammar (including insult and humor); (3) supplying neurobiological, ontogenetic, and clinical evidence of a link between (reactive) aggression and (reactive) verbal behavior; (4) identifying proxies of the earlier stages in evolution among cognitive disorders; and (5) identifying specific points of contact and mutual reinforcement between these two processes (self-domestication and early language evolution), including reduction in physical aggression and stress/tension, as well as sexual selection.

**Figure 1:**
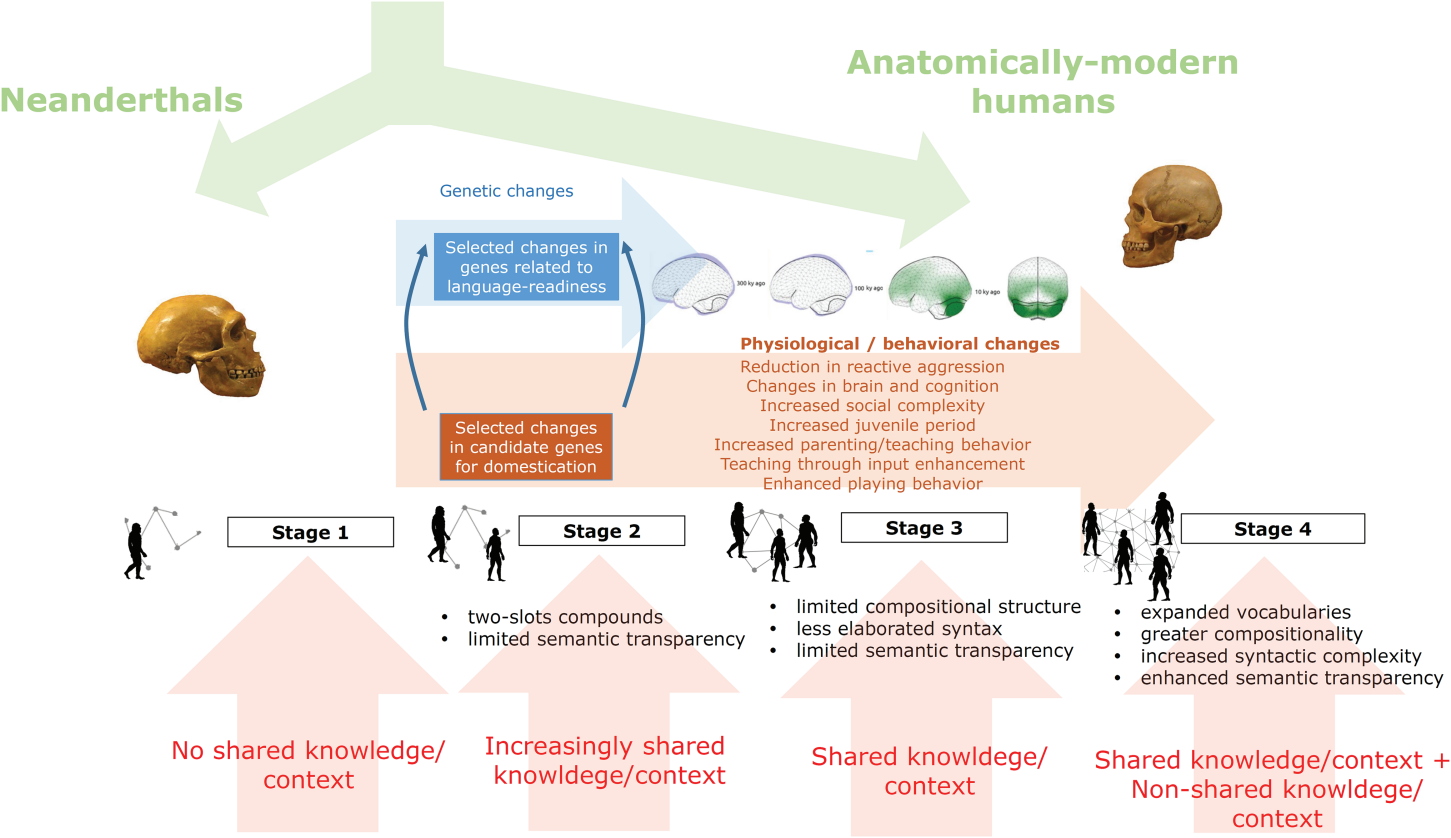
A graphical summary of the hypothesis of how languages might have changed with time in our species under the effect of our self-domestication.

One benefit of our proposal is that it helps solve the paradox of the two aggression types, reactive and proactive, which is raised by the Self-Domestication Hypothesis (SDH), that is, why proactive aggression has increased with time in spite of our increased self-domestication. The problem finds a direct solution in correlating early self-domestication processes with the emergence of simple forms of early language/grammar, featuring reactive verbal aggression; on the other hand, proactive aggression seems to be enabled in the later stages of self-domestication, which correlates with more complex forms of language (see Benítez-Burraco and Kempe, 2018; Kissel and Kim, 2019). The following stages outline our proposal (see also Figure 1):

1. *The first stage*, occurring roughly in the period prior to 200 kya, sees self-domestication processes only start to emerge, with reactive physical aggression still relatively high.
2. *The second stage*, occurring roughly from 200 to 50 kya, sees increased self-domestication favoring the emergence of early language forms with proto-grammars especially suitable for swearing and insult (i.e., reactive language), which began to gradually replace reactive physical aggression, serving the same function. This early language was insufficiently sophisticated to support proactive aggression. During this stage, there is an accelerated feedback loop between self-domestication processes and the solidification of the early forms of language, both promoting a reduction in reactive physical aggression.
3. *The third stage*, 50–10 kya (the Upper Paleolithic), saw self-domestication reach its peak. More cooperation and socialization and less reactive aggression created a niche for more complex forms of language and cognition.
4. *The fourth stage*, from 10 kya (the onset of the Neolithic period) to the present day, was characterized by even more complex language and cognition, which now affords the linguistic, cognitive, and cultural means (e.g., sophisticated weapons) for coordinating premediated, large-scale, proactive aggression^7^.

Our proposal regarding what characterized the second stage with respect to self-domestication and language evolution establishes a middle ground between two opposite but influential views, those of Chomsky and colleagues vs. Dediu and colleagues. On the one hand, based on their view of syntax/grammar as an undecomposable/unnegotiable block, Berwick and Chomsky (2011, 2016, also previous work) proposed that language/syntax emerged suddenly and recently, in its full complexity, “just a bit over 50,000 years ago” (Chomsky, 2005), with no possibility for any simpler stages or precursors, or “some 70,000–100,000 years ago, and does not seem to have undergone modification since then” (Bolhuis et al., 2014). On the other hand, based on the comparative evidence among *Homo heidelbergensis*’ descendants, Dediu and Levinson (2013) proposed that language dates back to at least *H. heidelbergensis*, to some 500–400 kya, suggesting that Neanderthals and Denisovans might have even spoken complex languages comparable to those of modern humans, which would imply hierarchical and recursive syntax. We therefore acknowledge that our proposed timelines may be subject to revision pending further evidence. By contrast, in our proposal, this time period (roughly 200–50 kya) was characterized by a pre-hierarchical stage of languages, to contrast with the hierarchical and recursive stage, which is often associated with Chomsky’s notion of Merge. To avoid potential terminological confusion, we will adopt the terms pre-hierarchical stage and hierarchical stage. While the pre-hierarchical stage is associated with rudimentary symmetrical, flat, and non-recursive grammars, the hierarchical stage is associated with the exact opposite: asymmetrical, hierarchical, (potentially) recursive syntax. Nonetheless, as will be discussed in the following section, there is continuity and common ground between the two types of grammar, with the pre-hierarchical stage directly foreshadowing the nature of the hierarchical stage itself.

Relevant to this debate is also the nature and significant degree of cross-linguistic variation attested in the expression of the hierarchical stage (e.g., transitivity and tense) among extant human languages. Considering this variation in detail led to the proposal that these widely diverging hierarchical solutions were a later add-on, superimposed upon the common proto-syntactic foundation, and that the hierarchical layers of language may not have emerged only once and uniformly (in Africa) along with our species, but instead multiple times, and independently, either within Africa, or after the dispersion from Africa, plausibly in response to cultural pressures and innovations (Progovac, 2015, 2016)^8^. At least under the uniregional view of human origins, this would argue against hierarchical syntax emerging earlier than around 100–50 kya with humans^9^.

## Emergence of Proto-Syntax and Verbal Aggression (Insult)

The use of profanity is characterized as “reactive language” (Bergen, 2016, p. 88) in the sense that it is typically impulsive and spontaneous, often referred to as automatic speech, or automatisms in aphasic studies (e.g., Jackson, 1884; Code, 2011). It contrasts with “intentional” language, which gets impaired in Broca’s and global aphasias and which is more complex, demanding greater working memory. We contend that reactive language (e.g., swearing) is continuous with reactive physical aggression, having gradually replaced the latter, during the second stage (roughly from 200 to 50 kya).

Consider the following verb-noun compounds (1–3) from English, Serbian, and Twi, collected from various sources, old and new (English and Serbian examples are from Progovac, 2015; Twi examples are from Kingsley Okai, p.c., 2011)^10^. This type of compound is found across a variety of related and unrelated languages, with similar imagery across cultures (for specific examples and further references, see Progovac, 2015)^11^. It is of note that compounds like these have transient lives – they get created, and then the vast majority of them get abandoned, with only few surviving. As a result, different generations of speakers will be familiar with different compounds on these lists, taken directly from Progovac (2016, p. 8; further data can be found in Progovac and Locke, 2009 and Progovac, 2015). The significance of these compounds is also that they specialize for insult when referring to humans, in a variety of languages, reinforcing our view that simplest grammars are especially suitable for insult. There is no other grammatical strategy that we are aware of that specializes for insult, and certainly not a strategy with so many tokens.

1. kill-joy, turn-skin (traitor), hunch-back, wag-tail, tattle-tale, scatter-brain, cut-throat, cry-baby, fill-belly (glutton), crake-bone (crack-bone), fuck-ass, fuck-head
2. cepi-dlaka “split-hair” (hair-splitter); guli-koža “peel-skin” (who rips you off); vrti-guz “spin-butt” (restless person, fidget); muti-voda “muddy-water” (trouble-maker); jebi-vetar “fuck-wind” (charlatan); vuci-guz “drag-butt” (slow-moving person); kosi-noga “skew-leg” (person who limps); podvi-rep “fold-tail” (one who is crestfallen); liz-guz “lick-butt”; poj-kurić “sing-dick” (womanizer)
3. Atoto-botom “dip-pocket” (pickpocket); kukru-bin “roll-dung” (beetle); nom-mmogya “suck-blood” (vampire); wodi-nii “kill-person” (killer)

These creations specialize for derogatory reference and can be quite obscene and cruel. They are also humorous and creative, especially considering the simplicity of their structure. These compounds are coined by one single (non-recursive) operation merging just one verb and one noun (for illustration, see below; for further evidence and a discussion of alternative views, and possible variation across languages, see Progovac, 2015, 2019). Predominating among them are concrete, basic nouns, and verbs, depicting body parts and functions^12^. However, this rudimentary compounding strategy can produce stunning new concepts, often abstract. Their high imageability and coarseness contribute to the strong visceral effect. As metaphors, they demonstrate the cognitive innovations important for language, in particular, our ability to transcend the signature limits of core knowledge systems and ultimately, to combine and unify conceptual units that belong to distinct domains (see Boeckx and Benítez-Burraco, 2014a for discussion). This enables us to metaphorize and metonymize, as well as to grammaticalize, and ultimately, to make languages change (see Benítez-Burraco, 2017 for discussion). They are thus also directly relevant for the consideration of cross-modality in the evolution of language, as discussed by, e.g., Cuskley and Kirby (2013) and Miyagawa et al. (2018).

These compounds exhibit features consistent with the primitive stages of language: grammatical simplicity; basic, concrete vocabulary; strong visceral effect; significant creativity; ability to transcend modalities to create new, abstract concepts (metaphoricity); ability to entertain and amuse (including humor); and continuity with complex syntax (see below). While these creations cannot be completely identical to what was created early in evolution^13^, they can serve as excellent proxies, or approximations, which can be used to test current hypotheses, as well as to formulate new ones. Moreover, even though they certainly fall short of modern syntactic riches, they provide the foundation, the template from which to build modern syntaxes, as discussed below. As argued by, e.g., Gil (2005), such simple (associational) grammars are sufficient for many practical purposes.

While it is hard not to be distracted (or disturbed) by their extraordinary content, it is necessary to focus on the grammatical properties of these compounds. It is because of the most rudimentary nature of their grammar that these compounds qualify as approximations/proxies of proto-syntax. One concrete consequence of this type of two-slot grammar (which can only fit one verb and one noun, e.g., *turn-skin*) is that it cannot depict transitive events, which would require (at least) three slots (e.g., *snake-turn-skin; or *snake-shed-skin). In fact, this type of grammar is incapable of distinguishing subjects from objects (for further evidence, see Progovac, 2015). The noun in these compounds can be either subject-like (*cry-baby; rattle-snake; turn-table; tumble-weed*) or object-like (*turn-coat/skin; tumble-dung* (beetle); *fold-tail; split-hair*), and sometimes, it is hard to tell (e.g., *scatter-brain; busy-body; dare-devil*). Moreover, unlike their hierarchical counterparts in (4), verb-noun compounds in English (or Serbian) are not recursive (5), in the sense that the output of one binary operation of V + N (creating another N, *pick-pocket*) cannot serve as input to another binary operation, combining, e.g., the verb (V) *chase* with the noun (N) *pick-pocket*.

4. truck-driver chaser (the one who chases those who drive trucks)
5. *chase-pick-pocket (the one who chases those who pick pockets)

This simple binary stage of language directly foreshadows the nature of modern grammars in two important respects. First, modern grammars (with their so-called Merge operation) are considered to be binary, too, creating structures in a pair-wise fashion. Second, modern grammars rely heavily on nouns and verbs to express predications, i.e., who (noun) does what (verb). One can certainly imagine different kinds of grammars (ternary, n-ary), and different vocabularies depicting totally different concepts and relations, but modern human grammars are designed in a painstakingly binary fashion, and mostly for the purposes of expressing who does what (to whom). Such noun-verb combinations are at the bottom and the beginning of almost every modern sentence^14^. In fact, syntactic theory (e.g., Minimalism and predecessors) considers that every sentence unfolds from this bottom layer, which typically features a merge of just one verb and one noun (phrase), resulting in a Verb Phrase (VP) or Small Clause (SC), as given in (6). This analysis of the modern sentence is one of the most insightful and stable postulates in this theoretical framework. It was originally outlined by Burzio (1981), Stowell (1981), and Kitagawa (1985) and further solidified in the work of Koopman and Sportiche (1991), Chomsky (1995), Adger (2003), Citko (2011), and many others. This merge operation at the bottom of the hierarchy is sometimes referred to as First Merge in syntactic literature (e.g., Adger, 2003), whereas Second Merge adds a second argument (subject), in another, higher layer/segment of the verb phrase, which may be referred to as little vP (where TP is the Tense Phrase, responsible for the expression of tense and finiteness):

6. TP > vP > SC/VP^15^

If one intends to express both a subject and an object, together with the verb (7), one cannot do so with just a single binary operation (note that human grammars do not seem to allow ternary merges, but only binary ones; e.g., Kayne, 1984). This now requires first assembling an intransitive verb phrase or VP (a verb and one noun) (8a), and then taking that VP as an assembled unit and merging it with another layer of structure, little vP (8b). And the same is true for expressing tense/time – yet another layer, TP, is added for that purpose (8c). But, importantly, at the bottom of both transitive (7,8) and intransitive (9,10) sentences lies the binary, and possibly flat, small clause combination of just one verb and one noun (phrase)^16^.

7. Petra will rattle snakes.
8. a. [_SC/VP_ rattle snakes] →
  b. [_vP_ Petra [_SC/VP_ rattle snakes]] →
  c. [_TP_ Petra will [_vP_ Petra [_SC/VP_ rattle snakes]]]
9. Snakes will rattle./Petra will rattle.
10. a. [_SC/VP_ rattle snakes] →[_SC/VP_ rattle Petra] →
  b. [_TP_ Snakes will [_SC/VP_ rattle snakes]][_TP_ Petra will [_SC/VP_ rattle Petra]]

The cross-out notation indicates the initial, underlying position of the subject in the small clause, before it moves to the TP layer. The Move operation in modern syntax can be seen as a strategy for connecting various layers of structure and, in this case, transforming the ancestral small clause into a full-fledged modern sentence. This certainly looks like a tortured, roundabout way to simply express who does what to whom. But this step-by-step building of modern syntactic layers, including transitivity, makes perfect sense if the original proto-grammar was preserved as the foundation upon which to build further layers, rather than discarded. It would have been a solid, sturdy step from which to reach for ever higher but shakier steps. The less plausible alternative would have been to step down first, to the platform with no grammar at all, and then start from scratch, trying to jump straight to the higher realms. But this would have resulted in very different modern grammars^17^. It is this binary requirement on building syntactic structure, already foreshadowed in the proto-grammar stage, that forces the hierarchy/asymmetry, which characterizes modern languages.

Even though our focus here is on verbal aggression and insult, the benefits of this kind of proto-grammar would have been much broader. This type of proto-grammar would have also provided a convenient strategy for naming animals [*tumble-dung; swish-tail* (*bird*)*; stink-bug*], plants (*tumble-weed; catch-fly*), objects, and places, as well as for expressing basic commands (e.g., *Catch fly! Turn snake!*) and statements (e.g., *Bug stink; Monkey see*), not to mention enhanced ways of thinking, because it enables one to create new concepts by merging two pre-existing concepts. They could have also been used for affective purposes between partners or for calming and comforting one’s children (e.g., *Baby sleep*), also contributing to alleviating stress and tensions. In addition, according to Progovac (2015, 2016), an important extension of this two-*word* proto-grammar would have been two-*clause* symmetric combinations involving binary formulae (typically AB AC), often expressing wisdoms and observations (e.g., *You seek, you find; You sow, you reap; Easy come, easy go; Come one, come all; First come, first serve(d); Like father, like son; Monkey see, monkey do*)^18^. Such symmetric/paratactic clause combinations, where clauses stand next to each other, would have foreshadowed modern-day subordination/hypotaxis, where clauses get embedded one within another (e.g., *Those who seek will find*.).

In summary, the postulated approximations of proto-grammar provide continuity with modern syntax in two essential ways: (1) in their binary nature and (2) in their reliance on noun-like and verb-like elements to express predication. Even though it has become customary to reduce syntax to Chomsky’s Merge, it is important to emphasize here that the combinatorics of syntax is just one aspect of it, determining how many elements can merge at a time (binarity), and how many times (recursion), and in which manner (flat or hierarchical). Human syntax/language is also undoubtedly designed to express predication, i.e., to express who does what (to whom), by using primarily verbs and nouns. Importantly, the way syntax became complex is not in just any old random way, but in a way that helps express, with more precision, who does what to whom (and when, and where, and how, and why)^19^. In both of these respects (binary combinatorics, and the focus on who does what to whom), verb-noun compounds are an excellent stepping stone into modern syntax. Importantly for our purposes, the proto-grammar strategy behind these compounds not only provides continuity with complex syntax but also provides a more graceful transition from animal cognition, and particularly, from animal behavior, i.e., their emotional vocalizations, to human behavior, *via* verbal aggression.

## Neurobiology of Physical Aggression and Reactive Language

The limbic system (a group of brain structures supporting emotion, motivation, and long-term memory; see Rolls, 2015 for review), the striatal regions, and parts of the cortex, particularly, the frontal and the temporal cortices (Dolan et al., 2002; Yang et al., 2009; Boccardi et al., 2011) support aggressive behavior. Highly aggressive subjects exhibit enlargement and atypical activation of striatal regions (particularly, the caudate; Gatzke-Kopp et al., 2009; Ducharme et al., 2012; Yang et al., 2017). The striatum has been associated with the dopamine system that governs the regulation of motivated behavior (Mogenson et al., 1980), and which is critically involved in the expression of aggression in animals (Rodriguiz et al., 2004), but it is also crucially involved in language processing (e.g., Krishnan et al., 2016; Viñas-Guasch and Wu, 2017). Domesticated rats exhibit size reductions of the striatal area (Kruska and Schott, 1977), and the limbic system exhibits the highest differences between domesticated animals and their wild conspecifics (reviewed by Kruska, 1988).

Similar brain areas are involved in both reactive and proactive aggression; however, only the latter is associated with a thinner anterior cingulate cortex (Yang et al., 2017), a region involved in the regulation of emotions and social behavior including conflict monitoring and empathy (Devinsky et al., 1995; Botvinick, 2007). The cingulate gyrus, which is part of cingulate cortex, plays a key role in language processing, contributing to speech production *via* its connections with Broca’s area (Bernal et al., 2015). Compared to chimpanzees, bonobos (who are less aggressive) exhibit stronger links between the anterior cingulate gyrus and the amygdala, a pathway involved in the inhibition of aggression (Rilling et al., 2012). Likewise, Roth and Strüber (2009) found that reactive aggression is associated with smaller, less active frontal brain structures and amygdala hyperactivity, whereas proactive aggression correlates with reduced response of the amygdala and of cortical regions related to empathic and social behavior. Compared to chimps, bonobos also show an enlarged dorsal amygdala (Rilling et al., 2012). The amygdala is also implicated in the activation of the hypothalamic-pituitary-adrenal (HPA) axis through connections with the hypothalamus (Davis, 1997; Ledoux, 1998). The HPA axis is a major neuroendocrine system encompassing the hypothalamus, the pituitary gland, and the adrenal glands and regulating a great number of bodily functions. A reduced response of the HPA axis to stress has been observed in most domesticated animals (Kruska, 1988; Künzl and Sachser, 1999; Trut et al., 2009). With respect to aggression and cognitive functioning, reactive aggression in humans is associated with lower levels of goal-oriented inhibition and higher levels of flexibility, and proactive aggression is associated with higher levels of working memory (Hecht and Latzman, 2018)^20^.

In comparison to other forms of language, the processing of swear words/profanity entails more involvement of the basal ganglia, limbic structures, thalamus, and the right hemisphere (e.g., Code, 2005, 2011; Bergen, 2016). The basal ganglia (i.e., the striatal regions) and the limbic system are also highly implicated in physical aggression. Disorders, which result in uncontrolled swearing/profanity, typically involve a basal-limbic connection dysfunction (discussed further in section “Disorders”). Basal-limbic structures are phylogenetically old, and the aspects of human communication associated with them are considered to be ancient, too (Van Lancker and Cummings, 1999; Bradshaw, 2001; Bergen, 2016), a potentially controversial claim (although see also Lieberman, 2000, 2009 on the ancient nature of basal ganglia). In this respect, Code (2005, p. 317) suggests that these forms of language might represent fossilized clues to the evolutionary origins of human communication. With brain damage affecting inhibitory processes, primitive behaviors (e.g., verbal automatisms) can emerge from primitive regions. In fact, damage to language centers in the brain can obliterate most language but leave swearing and expletives intact (see section “Disorders” for more details).

Differential impairment of reactive language versus intentional language implies that they employ distinct neural bases/pathways (Bergen, 2016, p. 87). The circuit that supports reactive language (including profanity) is evolutionarily far older, dominated by the limbic system, responsible for generating emotions and motor impulses, where the basal ganglia regulates and selectively suppresses such impulses (Bergen, 2016, p. 95). In disorders, such as Tourette’s syndrome with coprolalia, there is a failure of this regulatory function of basal ganglia (see section “Disorders”). The relevance of basal ganglia for emotional speech processes, including such basic emotions as fear and disgust, is also established in the work of Paulmann et al. (2009) and Péron et al. (2013). Emotional vocalizations by other primates and mammals also seem to be supported by this kind of pathway, involving the limbic system and the basal ganglia (Robinson, 1967; see also Gruber and Grandjean, 2017), suggesting that emotional, profane language has some continuity with emotional vocalizations in other animals.

In natural use, expletives, especially those that are highly taboo, elicit strong physiological responses (including increased heart rate and sweating; Bergen, 2016). Such words are used for fundamental expression of deep emotion, including fear, pain, frustration, as well as for sex and violence (Code, 2005). The use of profanity is more common in men than in women (Jay, 1980, 1995; Van Lancker and Cummings, 1999, but see section “Aggression, Verbal Behavior, and Sexual Selection” for a possible challenge to this view), and this is true even in language disorders (Code, 1982, 2011; Jankovic and Rohaidy, 1987; Bergen, 2016). Considering that reactive physical aggression is more frequent in men than in women and that self-domestication was primarily subject to sexual selection (see section “Child Development”), this parallelism between physical and verbal aggression reinforces our hypothesis that verbal aggression acts as a proxy/replacement for reactive physical aggression.

Finally, expletive compounds can be highly humorous. One of the main functions of humor is to provide relief from stress and tension, *via* laughter and mirth (Berlyne, 1972; Meyer, 2000; Buijzen and Valkenburg, 2004). Humor serves as a natural stress antagonist in situations of trauma and stress, by decreasing cortisol levels (Vrticka et al., 2013; Bains et al., 2014). Typically, wild animals exhibit a more pronounced cortisol response to stress, compared to their domestic counterparts (Künzl and Sachser, 1999; Künzl et al., 2003; Zipser et al., 2014; Kaiser et al., 2015). As noted above, domestication is associated with a reduction in the function of the HPA axis (Naumenko and Belyaev, 1980; Kruska, 1988; Oskina, 1996; Künzl and Sachser, 1999; Trut et al., 2009). Humor engages a core network of cortical and subcortical structures, including the meso-cortico-limbic dopaminergic system and the amygdala (Vrticka et al., 2013). In addition, humor can often serve as a form of strong assertiveness bordering on aggression, especially in cases of teasing and insult (see section “Child Development”). We therefore argue that humor’s dual functions (i.e., stress reduction function and verbal aggression), and its reliance on limbic structures supports our proposition that early forms of language provided relief from stress and tension, as well as a (verbal) alternative to reactive aggression, and thus reinforced the effects of self-domestication.

## Disorders

Of particular relevance to our hypothesis are disorders that exhibit an imbalance between inhibition and disinhibition of verbal aggression. In this section, we consider certain disorders, which imply a dissociation between derogatory language and (more complex) referential language. Some of these conditions have a genetic basis, with candidate genes positively selected in our species.

### Tourette’s Syndrome and Coprophenomena

Tourette’s syndrome (TS) is a hereditary tic disorder affecting the basal ganglia and the basolateral amygdala and hippocampal formation, circuitry involved in social decision making (Albin, 2018). It is sometimes accompanied by involuntary obscene speech and derogatory comments (coprolalia). Less commonly, TS patients may also exhibit copropraxia, which involves involuntarily making obscene gestures (Jankovic and Rohaidy, 1987; Singer, 1997; Freeman et al., 2009; Bergen, 2016). Although these coprophenomena and the TS syndrome more generally remain poorly understood, brain imaging, neurophysiological, and post-mortem findings implicate the cortical-striatal-thalamocortical pathways in the etiopathology of TS (e.g., Mink, 2003; Singer, 2005; Singer and Minzer, 2005; Ganos et al., 2013). These pathways overlap with striatal-cortical networks implicated in physical aggression (as discussed above) and with the Broca’s-basal ganglia network essential for speech and language processing (e.g., Lieberman, 2000, 2009, 2015; Ullman, 2006). TS also tends to include repetitive involuntary eye, facial, and head movements, as well as explosive outbursts (Budman et al., 2008; Kano et al., 2008; Chen et al., 2013; Ganos et al., 2014). Given that the major functional role of eye, face, and head movements is social signaling, Albin (2018) suggested that the coprophenomena associated with TS may be best understood as distortions of reactive, spontaneous social signals, thus possibly implicating the brain areas involved in TS in the evolution of early language. The use of foul reactive language at the early stages of human self-domestication may have strengthened these brain circuits, easing the way into more complex forms of language^21^.

Patients with TS experience an increase in their tics under stressful conditions, which are accompanied by a sense of discomfort that is relieved by tic performance (e.g., Cohen and Leckman, 1992; Leckman and Peterson, 1993; Evers and van de Wetering, 1994; Jankovic, 1997; Banaschewski et al., 2003; Kwak et al., 2003; Woods et al., 2005; Corbett et al., 2008; Albin, 2018). Importantly, a subset of TS patients exhibits heightened reactivity to stress of the HPA axis (Chappell et al., 1994). Likewise, children with TS show higher cortisol levels in response to stressors, which are indicative of an enhanced HPA responsivity to stress (Corbett et al., 2008). This is relevant to the self-domestication hypothesis of human evolution, because, as noted above, domestication entails reduced response of the HPA axis to stress. In this respect, TS can be seen as exhibiting attenuated features of self-domestication, positing an intriguing parallelism with autism, also proposed to exhibit some features of a less-domesticated phenotype (Benítez-Burraco et al., 2016).

Rare mutations in selected genes have been identified in some TS patients. One of these genes is *SLITRK1*, which encodes an integral membrane protein involved in neurite outgrowth (Miranda et al., 2009). *SLITRK1* has an evolutionarily conserved expression pattern in projection neurons of the corticostriatal-thalamocortical circuits and cortical pyramidal neurons, contributing to the development of connections between the cortex, the striatum, and the thalamus (Stillman et al., 2009). Incidentally, there is also an ancestral mutation of *SLITRK1* (S330A) that has been related to TS (Ozomaro et al., 2013; Alexander et al., 2016). This SNP is highlighted by Theofanopoulou et al. (2017b) as a sort of window to the “underdomesticated” phenotypes found in other hominins. Overall, these genetic findings suggest that TS is more related to ancestral genomic variants than to derived changes in modern humans.

### Aphasia and Speech Automatisms

Aphasias, resulting from brain damage, involve disinhibition of speech automatisms, such as counting, rhyming, prayer, but most commonly expletives and modal/auxiliary sentence stem structures (e.g., *I cannot*; *I try*; Code, 2005, 2011; Code et al., 2009). These two most frequent subtypes are also most relevant for evolutionary considerations. For the severest cases of non-fluent aphasia, these automatisms may be the only speech produced (Code, 2011, p. 139). Speaking specifically about derogatory language, Code (2011) points out that naturally occurring expletives emerge from ancient areas of the limbic system (see also Code, 1987; Leckman et al., 1991; Speedie et al., 1993; Van Lancker and Cummings, 1999). On the other hand, in pathology, expletives seem to emerge from disinhibited basal-limbic structures, which are normally under control from prefrontal networks, where basal ganglia damage appears to be essential for the production of an aphasic automatism (Brunner et al., 1982). With aphasias, we witness a loss of the complex compositional language, while the reactive, derogatory language is preserved. According to the so-called last in, first out principle (see e.g., Code, 2005; also Gibson, 2009), what is acquired last is the most shallow/fragile layer that is the easiest to lose, and vice versa. In other words, the most recently evolved components of cognition, which certainly include compositional language, are the least robust, and most prone to damage and loss. If true, this provides further evidence of the role of reactive verbal aggression in language evolution.

This raises the question of whether the production of automatisms is associated with a higher degree of stress, and whether such production helps relieve stress. While there are many reports to the effect that aphasics in general experience a lot of stress and anxiety, even anger, specifically in trying to use language (see e.g., Goldstein, 1942; Luria, 1970; Laures-Gore et al., 2007; Cahana-Amitay et al., 2011; Laures-Gore, 2012), we have not come across any reports addressing specifically the production of automatisms in this respect. It would be of interest for future research to determine whether or not the incidence of specifically cursing and derogatory automatisms correlates with the experience of higher stress and anger (and thus higher cortisol levels), as well as whether the uttering of such automatisms helps relieve stress, in a way comparable to the production of tics in TS (section “Tourette’s Syndrome and Coprophenomena”).

In summary, our discussion of language/cognitive disorders in relation to self-domestication and language evolution supports the view that these disorders can inform on aspects of human domestication. They, moreover, involve patterns of inhibition and disinhibition that seem to be just poles on the continuum of cognitive modes, encompassing also the typically developing cognition. The discussion of disorders also highlights the existence of significant individual variability across all the dimensions relevant for language processing, which, moreover, seems to be genetically influenced. These considerations suggest that the evolution of language cannot be a simple, straightforward step, but rather a complex, multi-faceted, and multi-gene phenomenon, recruiting and coordinating a variety of cognitive systems and functions, with each new development potentially subject to genetic and/or cultural evolution.

## Child Development

While ontogeny does not literally recapitulate phylogeny, there are usually points of comparison (e.g., Ridley, 1993). Here we report on some notable parallels between childhood development and our model of language evolution, with a focus on aggression, verbal (derogatory) behavior, and complex language. First, in the transition from infancy to childhood, when syntax emerges, there are developments in three other relevant areas: the ability to spontaneously coin compounds (Becker, 1994); the tendency to tease and insult, and thus, the onset of humor (McGhee, 1976; Apte, 1985); and the onset of agonistic verbal engagement or verbal dueling (Gossen, 1976; Wyatt, 1995, 1999). Second, as noted by these and other authors, teasing and insulting, as well as verbal dueling, tend to predominate in males, even at the time of their appearance in late infancy or early childhood, pointing to the relevance of sexual selection, and providing further supporting evidence for our proposal.

Regarding the emergence of syntax, children use simpler/simplified syntactic structures early on, and combinations of just one verb and one noun (intransitive structures) predominate in early child grammars cross-linguistically. It is beyond the scope of this paper to get into different types of theories and controversies behind these omissions/simplifications, as the literature on this topic is vast and varied. Suffice it to note here that, at least on the surface, early children grammars often express only one noun argument per verb (see e.g., Zheng and Goldin-Meadow, 2002; Rakhlin and Progovac, 2017). Children’s early utterances also include novel compounds of various kinds, including noun-noun and verb-noun combinations, for example, light-man (electrician); nose-beard (whiskers); and push-ball (a ball for pushing and bouncing; Becker, 1994). Compounding of this type seems to be a rather simple, straightforward strategy for children expressing new concepts.

There are also experiments targeting specifically compounds using verbs and nouns, establishing a clear difference in the order and ease of acquisition between flat verb-noun compounds and their hierarchical counterparts. In their experiment, Clark et al. (1986) prompted children to produce hierarchical -*er* compounds (e.g., *This is a cheese-grater*; *paper-ripper; ball-bouncer*). At around three, instead of these targeted compounds, children consistently produced related verb-noun combinations (i.e., *This is a grate-cheese; rip-paper; bounce-ball*). Before reaching the target adult-like stage, many children also experienced another stage, where they produced compounds with misplaced affixes (i.e., *This is dry-hair-er/dry-er-hair* in lieu of *hair-dry-er*) or (*This is a fix-bik-er/fix-er-bike* in lieu of *bike-fix-er*).

Some conclusions from child language studies are important for our hypothesis. First, the stages and struggles in the acquisition of these compounds reinforce the view that -*er* compounds are related to VN compounds, as both rely on the common foundation provided by the flat (paratactic) verb-noun composition. Second, children start with the simpler structures, with the foundation, before they can scaffold to the hierarchical supra-structure, as emphasized by Clark et al. (1986). Third, VN compositions seem to be more primary and simpler than their hierarchical relatives.

With regard to the second area of development, namely, the onset of humor (and the tendency to tease and insult), laughter is one of the first social vocalizations in human infants, with an early onset at approximately 4 months of age (Ruch and Ekman, 2001). Responsive smiling generally develops even earlier, within the first 5 weeks (Kraemer et al., 1999). The earliest form of humor in young children, incongruity-based humor, relies on principles of discrepancy applied to actions, such as clowning and acting silly (McGhee, 1976). This kind of humor has also been reported for other primates (Patterson and Gordon, 1993). McGhee also reports a gender difference emerging at the age of 6–11 years old, but not before that. Specifically, he found that boys laughed more frequently than girls (the girls instead tended to smile), that they initiated humor more often, whether by non-verbal or verbal means, and that they also showed more hostility in their laughter and humor, including ridicule and insult. McGhee concluded that attempts to initiate humor or laughter in the presence of others can be seen as a form of strong assertiveness, especially in the case of hostile humor. This is directly relevant for our hypothesis of verbal aggression (partly) replacing physical aggression, which also predominates in males.

Finally, concerning the third area of development that we wish to highlight (the onset of agonistic verbal engagement or verbal dueling), it has been found that, cross-culturally, boys aged 3–11 engage in rough and tumble play, as well as verbal aggression, significantly more than do girls (Whiting and Edwards, 1973; Apte, 1985, p. 71; but see Björkqvist, 2018, for a possibly different view). Likewise, in many cultures, adolescent boys and men tend to engage in ritual insults (e.g., Apte, 1985, p. 70). Marsh (1978) provides convincing evidence from a variety of situations and cultures that ritual insult exchanges often serve instead of physical violence. This is consistent with our view that verbal aggression provides a different channel to the same goal, involving less risk of physical harm, thus contributing to better survival.

## Aggression, Verbal Behavior, and Sexual Selection

Self-domestication in humans has been attributed to sexually selective forces, including selection against (physical) aggression, and in favor of pair-bonding beneficial for child rearing (Hare et al., 2012; Stanyon and Bigoni, 2014; Okanoya, 2015; Gleeson, 2018). Likewise, the emergence of early grammars, especially suited to verbal aggression (insult), has been attributed to sexual selection for creative cognitive abilities (Progovac and Locke, 2009; Progovac, 2015). Furthermore, the use of both verbal and physical aggression seems more prevalent in males, revealing a dimorphism characteristic of sexual selection. Starting early on in childhood, and continuing into adulthood, across a variety of cultures, both physical aggression and verbal aggression show significant gender differences in favor of males (Whiting and Edwards, 1973; Apte, 1985), including with language disorders (Code, 1982, 2011; Jankovic and Rohaidy, 1987; Bergen, 2016). This gender discrepancy in both types of aggression suggests that they cluster together and that they have a common underlying cause, consistent with our proposal that verbal aggression served to replace (reactive) physical aggression.

Franks and Rigby (2005) observed that men increase their creativity with language in the presence of both attractive women and male competitors. Creativity is highly correlated with intelligence (Miller, 2000), implicating creative language use in both mate attraction and intra-sexual competition in men. Furthermore, eloquent speakers tend to be granted the highest social status (Tallerman, 2013, p. 95), which in turn is correlated with greater reproductive success (Locke, 2009). Following Gleeson (2018, p. 8), we contend that any increase in language complexity may imply selection forces favoring such complexity (see Progovac, 2019), directly implicating sexual selection in the proliferation of more complex, creative language.

Furthermore, while sexual dimorphism has decreased in humans during the period of self-domestication, it has certainly not been eliminated. In his review article, Gleeson (2018) makes a case for the relevance of sexual selection in the evolution of humans, and he observes that female preferences must have been for moderately masculine males, rather than for extremely non-masculine (domesticated) ones, likely reflecting conflicting forces in sexual selection^22^. On the one hand, there are female preferences for male investment in pair-bonding, but on the other hand, there are also female preferences for physically stronger, more masculine males, which seem to be context-dependent, and to vary relative to environmental and other circumstances, related to survival (Trivers, 1972; Kruger, 2006; Archer, 2009; Quist et al., 2012). Boothroyd et al. (2017) found that moderately masculine fathers had more surviving offspring than those with both relatively low and relatively high masculinity, suggesting a centralized optimum of masculinity. It is also worth observing that some indicators of masculinity have infiltrated language, including low vocal pitch, as well as the initiation of humor, often analyzed as building and then resolving tension/incongruity, and considered by McGhee (1976) to reveal strong assertiveness, especially given that it involves a risk of failure. Both of these features seem to be subject to female preferences, possibly indirectly contributing to the preservation of (moderate) masculinity.

Furthermore, males exhibit displays of physical prowess to the formidability of male competitors, as well as characteristics such as facial hair and low vocal pitch, that increase perceptions of dominance (Hill et al., 2017). These traits are of direct relevance for sexual selection because they show sexual dimorphism, they emerge around puberty, and they correlate with success in mating and reproduction. Importantly, the specific derogatory compounds, which we argue are reflective of early language, are illustrative of both inter- and intra-sexual selection. Regarding male to male competition, these compounds often describe men in derogatory terms, but even when they seemingly describe women, such compounds are still typically used to derogate men, for a doubly insulting effect (Mihajlović, 1992; Progovac and Locke, 2009)^23^. As pointed out by Marsh (1978), the most frequent type of insult among men even today has to do with emasculating one’s opponent. Their usefulness in derogating existing rivals and placing prospective rivals on notice (aggressive rivalry), and in demonstrating verbal skills, humor, and quick wittedness simultaneously engages both sides of the sexual selection equation (Progovac and Locke, 2009). Such verbal items would have afforded a particularly useful, low-risk (non-violent) way to compete for status and sex. Of direct relevance for our proposal is Hill et al.’s (2017) conclusion that intra-sexual selection led to enhanced same-sex intimidation, or formidability, instead of actual combat. In this respect, derogatory language can be viewed as the most innovative and creative means of achieving such “formidability,” which straddles the boundary between physical and cognitive strength.

According to Card et al.’s (2008) meta-analytic review of 148 studies, there exist clear gender discrepancies favoring boys in direct (reactive) aggression, and only trivial differences favoring girls in indirect aggression (see also Björkqvist, 2018). While Björkqvist (2018) suggests that boys and girls are equally aggressive when it comes to verbal aggression, the evidence for this claim is not provided in this opinion piece, and it contradicts many reports which have found such a difference favoring males in verbal aggression, whether with typical populations [section “Neurobiology of Physical Aggression and Reactive Language”], or impaired populations (section “Disorders”). While reactive physical aggression in humans has seen a decline, as discussed at length in the previous sections, it still exists, and it (still) shows a prominent gender difference. According to, e.g., Archer (2009), the extent and the nature of gender differences in aggression can be better explained by sexual selection, given that they increase with the degree of associated risk, occur early in life, and peak in young adulthood.

There are also gender differences in initiating and perceiving humor. Adolescent and adult females exhibit greater emotional reactivity during humor perception than do males (Vrticka et al., 2013). This supports the fitness indicator hypothesis of humor, related to female preferences. Unlike with humor appreciation, where striatal activation follows or coincides with activation of temporal regions, with humor creation (which exhibits a male bias), the peak striatal activation precedes the peak of temporal activation (Amir and Biederman, 2016). The striatum (basal ganglia) is also implicated in both physical and verbal aggression. Both types of gender differences, those associated with the initiation of humor, and those associated with the appreciation of humor, directly implicate sexual selection in the feedback loop that we propose was critical to the evolution of language and self-domestication.

Three hormones were likely targets for sexual selection with respect to a reduction in physically aggressive behavior: serotonin, testosterone, and oxytocin (Kuepper et al., 2010; Montoya et al., 2012). Low testosterone has been related to male prosociality and parental care (Burnham, 2007). Exogenous serotonin increases harm avoidance and cooperative behavior (Wood et al., 2006; Crockett et al., 2010) and increases in brain levels of serotonin correlate with reduced emotional reactivity and aggression in experimental animal populations selected for friendliness toward humans (Plyusnina et al., 1991; Agnvall et al., 2015). In domesticated animals and bonobos, an increase in serotonin and a reduction in testosterone are associated with facial feminization and reduced cranial capacity (Hare et al., 2012). Although archaic human species had similar sized brains compared to *H. sapiens*, their faces seem to be more masculinized than the oldest modern humans (Churchill, 2014; Hare, 2017). It is also relevant that changes in the brain seem to have predated changes in our face morphology, possibly because of our mild self-domestication at that initial stage. Finally, oxytocin has been claimed to modulate the multimodality that characterizes higher-order linguistic abilities, including the vocal-auditory system, the attentional-memory system, and the socio-interactive system (Theofanopoulou, 2016) because of its regulatory role on the development of specific neural pathways (e.g., Theofanopoulou et al., 2017a on vocal learning).

We thus conclude that sexual selection of self-domestication interacts with sexual selection for verbal aggression, possibly in conflicting ways, which may account for the complicated picture of the expressions of masculinity described above: while the former favored less physically aggressive males, the latter favored verbal behavior/aggression, which, at early stages of language emergence, brought about novelty, creativity, and verbal humor. The net result would converge on selecting those who are not just less aggressive, but who are also better able to use verbal aggression to replace physical aggression, as they would be selected by both processes. This contrasts with the conclusion reached by Stanyon and Bigoni (2014), who argue that it was reduced male competition and increased female choice that favored cognitive evolution. While this is certainly one part of the story, our proposal implies that the continued male competition in the realm of verbal aggression/verbal behavior also contributed substantially to the evolution of cognitive abilities, at least at this early but crucial step in the emergence of language and evolution of self-domestication.

## Discussion and Conclusions

Here we proposed that that self-domestication favored the emergence of a phenotype prone to replace reactive physical aggression with verbal aggression. The (partial) transition to verbal aggression and verbal behavior more generally then favored self-domestication, *via* a mutually reinforcing feedback loop, since verbal behavior affords less violence, better survival, and more opportunities for social interactions, ultimately paving the way for the evolution of more complex forms of language. We further proposed that looking at the (gradual) emergence of verbal means of aggression (approximated by proto-grammatical compounds) helps illuminate the initial steps of the language evolution/self-domestication feedback loop. The novelty of our approach lies in (1) giving an active role to early forms of language in interacting with self-domestication processes; (2) providing specific details and functions of this early stage of grammar (including creative uses of insult and humor); (3) supplying neurobiological, ontogenetic, and clinical evidence of a link between (reactive) aggression and (reactive) verbal behavior; (4) identifying proxies of the earlier stages in evolution among cognitive disorders; and (5) identifying specific points of contact and mutual reinforcement between these two processes (self-domestication and early language evolution), including reduction in physical aggression and stress/tension, as well as sexual selection.

One immediate advantage of our proposal is that, as noted, it helps solve the paradox of the two aggression types, reactive and proactive, which the Self-Domestication Hypothesis (SDH) on its own cannot solve. If SDH simply postulates that humans were selected for their friendliness and lack of aggression, then this discrepancy between the two aggression types is unexpected. But the problem finds a direct solution in correlating early self-domestication processes with the emergence of simple forms of early language/grammar, as per our proposal in this paper, but also in correlating later stages of self-domestication with more complex forms of language, as discussed by Benítez-Burraco and Kempe (2018) and Kissel and Kim (2019). Given that the postulated proto-grammar is particularly suitable for expressing crude and often obscene insults, representing essentially reactive language, this kind of language would have been most useful in countering/replacing reactive aggression, but as such, it would not have affected any existing or emerging proactive aggression.

**Figure 2:**
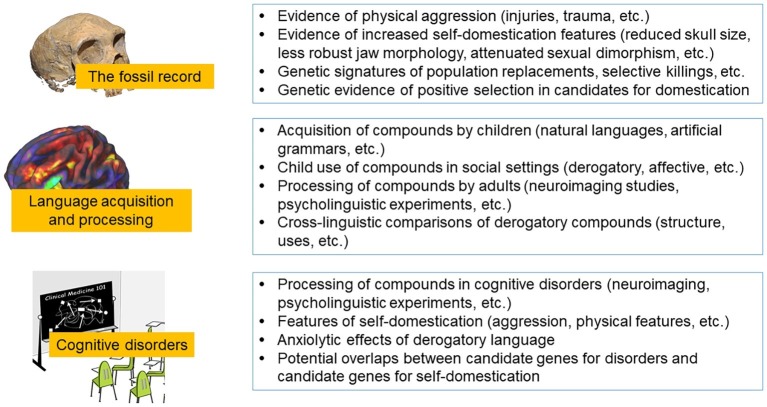
Testing arenas for the most relevant predictions resulting from the hypothesis discussed in the paper. Image attribution: Above: “File:Neanderthal skull from Forbes’ Quarry.jpg” by AquilaGib is licensed under CC BY-SA 3.0. Middle: “fMRI Image of Preteen Brain” by National Institutes of Health (NIH) is licensed under CC BY-NC 2.0. Below “File:Clinical Medicine 101 – journal.pmed.0020111.g001.png” by Daniel Mietchen is licensed under CC BY 2.5.

Several classes of predictions arise from our proposal, yielding specific hypotheses. We single out three such classes: (1) the history of aggression and the fossil record; (2) linguistic proxies (fossils) of the second (proto-grammar) stage in (language) evolution, and their acquisition and processing implications; and (3) Disorders and (verbal) aggression. For each of these classes, we identify some specific hypotheses that are subject to testing and falsification (see also Figure 2).

1. The history of aggression and the fossil record.
  - First, we predict a gradual decrease in reactive physical aggression, accelerated during especially the second and third stages, but also continuing into the present times. This scenario already seems well supported (see e.g., Cieri et al., 2014 for the claim that features of self-domestication reached a peak at the end of Upper Paleolithic). Still, this is a hypothesis in need of further testing.
  - Second, we predict an increase in proactive aggression starting in the third stage, and accelerating in the fourth stage, consistent with the considerations of gradual language evolution. There is already some initial evidence for this hypothesis, as collaborative inter-group conflicts became widespread during the Neolithic (Zeng et al., 2018). But further evidence can certainly be sought to better support or falsify this hypothesis. For example, evidence of accelerated proactive aggression in the first or second stages postulated above would falsify our hypothesis and would at least necessitate a reconsideration/revision of the timeline.
2. Linguistic proxies (fossils) of the second (proto-grammar) stage in human evolution.
  - Our first prediction is that the flatter evolutionary proxies will be acquired earlier by children, and with less effort, than their more hierarchical counterparts. As mentioned in Section “Child Development,” some experiments with children already established that what we refer to here as “fossil” compounds are acquired earlier, and with more ease, than their hierarchical counterparts (Clark et al., 1986). Such experiments can be replicated with additional language proxies and conducted using additional languages, or even by using artificial grammars.
  - Similar expectations hold for the processing of human language by adults, where the prediction is that the processing of flatter, fossil structures, such as small clauses and compounds, in contrast to their syntactically more layered counterparts, will rely less on the more recently enhanced brain networks. Progovac et al. (2018a,b) report some preliminary results of fMRI experiments along these lines that establish clear processing differences between the two types of structures, but more studies are needed to confirm or disconfirm these results, especially cross-linguistic studies, including a variety of languages. This line of research can help determine what kind of brains are needed for the (effortless) processing of early language vs. modern languages and would potentially tie into the considerations of the evolution of the human brain and the human skull, as discussed in section “Introduction.”
3. Disorders and (verbal) aggression.
  - The anxiolytic (stress and anxiety-relieving) properties of reactive verbal aggression are hypothesized to have contributed to the language emergence/self-domestication feedback loop. While there are proposals in the literature to the effect that tics in TS are anxiolytic (section “Tourette’s Syndrome and Coprophenomena”), this should be subjected to further experimental testing. We further predict that tics accompanied by coprolalia (uncontrollable profanity) will provide better stress relief than those without it.
  - We make a similar prediction when it comes to automatisms in aphasia. The production of these automatisms, specifically expletives, seems to be associated with a higher degree of stress, and experiments can be designed to gauge whether such production is anxiolytic.

The truth is that very little is known about swearing and derogatory language, including its processing and genetic basis, whether in typical populations, or in disorders, most probably because this kind of language is often taboo, and typically avoided even in scientific research^24^. However, once tapped into, these phenomena, including the neuroscience and genetics of the functions and dysfunctions of swearing/derogatory language, will provide an especially fertile ground for formulating and testing a variety of hypotheses about language evolution and self-domestication, and human evolution more generally.

## Data Availability Statement

The datasets generated for this study are available on request to the corresponding author.

## Author Contributions

LP and AB-B conceived and wrote the manuscript.

### Conflict of Interest

The authors declare that the research was conducted in the absence of any commercial or financial relationships that could be construed as a potential conflict of interest.

## Footnotes

^1^These two processes are expected to have incurred in some sort of gradualism (which is not incompatible with occasional punctual sudden changes). In truth, gradual changes have been reported for the globularity of the human skull. According to Neubauer et al.’s (2018) study of endocranial casts, hominin fossils from first anatomically modern humans did not exhibit this type of globularity, in clear contrast to human skulls from more recent periods, which they found to be within the range of modern humans. They also identified an intermediate stage in the evolution of globularity, with the fossils dating somewhere between 130 and 100 kya. Overall, in the first step of this process, approximately 200–130 kya, the frontal area became taller, and the parietal areas and the cerebellum bulged and became larger, while the occipital area became more rounded. The second step, roughly 130–100 kya, involved size changes primarily in the cerebellar and occipital areas. Both steps contributed to the globular shape of the human skull. Neubauer et al. (2018) further suggested that the initial changes in the shape of the skull were caused by brain reorganization, rather than changes in the shape of the face, which are typically found in domesticated animals. Moreover, these morphological changes in the evolution of humans were not likely to have been a result of genetic drift, but rather of selection, given that there is evidence for positive selection of several genes expressed in brain development, including the genes responsible for axon and dendrite growth. Something similar occurs with our behavior, including our cultural practices. Based mostly on archeological findings, Mellars (2002) and others initially proposed that there was a major cultural and cognitive transition/revolution around 43–35 kya. However, later discoveries led to a revision of this view, pointing to a more protracted, gradual accretion of culture (see e.g., McBrearty and Brooks, 2000; McBrearty, 2007; Mellars, 2007, p. 3). Neubauer et al. (2018) further note that the globularization timeline parallels the emergence of behavioral modernity, culminating around 50–40 kya, suggesting that some of those genes expressed in brain development were selected and fixed. In summary, comparable to the morphology of the skull/brain, behavioral modernity also represents a culmination of gradual accretion over time, rather than a single rapid evolutionary event.

^2^Some differences exist between primate and human inter-group aggression, considering that humans usually form peaceful relationships and alliances among groups (see Wrangham and Glowacki, 2012 for discussion). Socially coordinated violence (proactive aggression) potentially became possible only with the onset of symbolic thought and complex cognition because the same capacities for communication and sociality allow warfare and conflict resolution and avoidance (Kissel and Kim, 2019). In fact, features of self-domestication reached a peak at the end of Upper Paleolithic (Cieri et al., 2014), right before collaborative inter-group conflicts became widespread during the Neolithic, as shown by genetic evidence (Zeng et al., 2018).

^3^While it may be true, as pointed out by a reviewer, that derogatory language can be used playfully in an endearing way, this is of course also true of physical aggression, such as hitting. This does not undermine the view that the primary function of both of these phenomena is aggression. As the reviewer also points out, complex language can certainly be used for derogatory purposes, even when seemingly polished and polite. This does not pose a problem for our approach, which associates such complex, pragmatically refined capabilities with the later developments in human evolution, which brought about more complex forms of language, with more sophisticated pragmatic skills, and, arguably, also more sophisticated tools for planning and coordinating proactive aggression.

^4^Sexual selection is also thought to be one important triggering factor of self-domestication features, with females selecting less aggressive males, as discussed in section “Aggression, Verbal Behavior, and Sexual Selection.”

^5^Künzl et al. (2003) found that long-term breeding and rearing of wild guinea pigs in captivity did not result in significant changes in behavior and hormonal stress responses in comparison to domestic guinea pigs. They concluded that it takes much longer periods of time, as well as artificial selection by humans, to bring about characteristics of domestication in wild animals. Human self-domestication did not involve artificial selection by others, and as such is not expected to have been instantaneous. Similar processes of slow self-domestication have also been observed in bonobos (Hare et al., 2012).

^6^Pending further evidence, this timeline, especially the starting point, has to be considered approximate. As pointed out by a reviewer, researchers have proposed that some forms of language with grammar may have been in place as early as 500 kya, based on the skeletal and genetic evidence from Neanderthals (e.g., Dediu and Levinson, 2013; see also Johansson, 2005; Zilhão, 2011).

^7^However, the picture is a bit more complicated. While there is a clear overall trend toward evolving self-domestication features in humans, including less masculinized traits in men, Cieri et al. (2014) found that Neolithic humans exhibit more masculinized features compared to Upper Paleolithic humans, as well as compared to present-day hunter-gatherers, attributing the effect to the more hierarchical and man-dominated nature of agricultural societies, where women have less opportunity to exert their sexual selection preferences, as compared to relatively egalitarian hunter-gatherer societies (see section “Aggression, Verbal Behavior, and Sexual Selection” for further discussion of these issues within a sexual selection scenario). It is also possible that proactive aggression contributed to this development, as it brings about wars, creating a new environment where stronger, more aggressive males would have been favored by both natural and sexual selection.

^8^According to, e.g., Stringer (2007) and Finlayson (2009), there are still many uncertainties about human timeline and dispersals. Stringer (2007, p. 17) mentions a possibility for an African version of multiregionalism, citing “growing molecular evidence of deep divisions within African populations” (see also Wong, 2017 for some recent findings). Under this scenario, hierarchical syntax could have emerged much earlier, independently among different populations in Africa, more in line with Dediu and Levinson’s (2013) view.

^9^It is of note that Chomsky (2005) has also advocated a rather late emergence of hierarchical syntax, around 50 kya, as pointed out in the text. The difference is that on his approach syntax emerged suddenly in all its complexity, and uniformly, without any precursors, while on our approach syntax evolved gradually, and often differently in different populations, with precursors that interacted with the domestication and other processes involving the evolution of the brain. Also, for Chomsky, the evolution of syntax was fully biological/genetic, while in our view, it involves a complex feedback loop between culture and genes.

^10^Weekley (1916) collected a sizeable number of English verb-noun compounds. According to him, this expressive way of naming, often exhibiting unquotable coarseness, flourished in thirteenth and fourteenth centuries, yielding thousands of tokens. Mihajlović (1992) collected over 500 Serbian people and place names in this form, reporting that these condensed compositions pack in them “frozen fairy tales, proverbs, and ancient wisdoms and metaphors” (Mihajlović 1992, p. 8,9). Darmesteter (1934, p. 443) was impressed by the “artistic beauty and richness” of such derogatory compounds in French.

^11^Contrary to Nóbrega and Miyagawa (2015)’s view, in order for such compounds to count as approximations (“fossils”) of early stages of syntax, they do not necessarily need to be found in every human language, with exactly the same characteristics (Progovac, 2019 offers a detailed defense of syntactic “fossils” in this sense). The claim is that this is the starting point, the bedrock upon which one can build (or not) various types of syntactic complexity, as discussed below. Different languages in fact offer different types of fossil structures in this sense, some of them rare to find across modern languages (as discussed by Progovac, 2015).

^12^See also Samarin (1969) for Gbeya insults, which also fixate on body parts and physical appearance. In fact, Mohr (2013) provides evidence that such vulgar expressions were completely appropriate to use in, e.g., Roman times, only 2 kya, clearly at the stage of advanced modernity. As she argues, the appropriateness of such language coincided with a much less strict sense of privacy in performing bodily functions, as well as in covering body parts with clothing. Whatever we might think of such language today, it played a much bigger role in ancient times.

^13^For example, while in modern languages, the categories of verbs and nouns are typically distinguished grammatically, this would not have been the case at the time when grammar/language just started emerging.

^14^It is also of note that Heine and Kuteva (2007) reconstructed a stage of human language evolution in which only nouns were used, followed by a stage in which both nouns and verbs were used, but no other categories, arguing that other categories gradually gammaticalize from nouns and verbs.

^15^The representation in (6) depicts the basic (partial) hierarchy of sentential structure, widely adopted in this syntactic framework. These are the least controversial layers, sufficient for our purposes, but there are certainly several others that have been postulated (see, e.g., Adger, 2003).

^16^It is of note that in some cases what counts as subject vs. object can get blurred, depending not so much on the noun’s inherent relationship with the verb, but more on whether or not there are additional noun arguments. This is the case with the noun *snake* in the two derivations in the text. This is relevant for the claim that this bottom layer by itself is not capable of distinguishing subjects from objects. The reader should also note that these derivations are simplified by omitting certain steps and null categories (such as null v head), which are not relevant for the discussion.

^17^One possibility would be n-ary (as opposed to binary) grammars, with certain designated slots with fixed ordering for tense, subject, verb, object, without grouping these categories into constituents and subconstituents, and without some of these categories exhibiting syntactic dominance over the others. One can also imagine languages that are not obsessed with who does what to whom. There seems to be nothing inevitable about evolving grammars with binary branching based on predication typically expressed by verbs and nouns.

^18^Such expressions are preserved much better in some languages than others (see Progovac, 2015, 2016 for discussion and references.) For a more theoretical discussion of the relevance of symmetry vs. asymmetry in human language and evolution, see also Citko (2011) and Progovac (2015).

^19^This is in fact where languages differ profoundly. There are several different strategies for discriminating between subjects/agents from objects/patients, including, but not limited to, ergative-absolutive grammars, split-ergative grammars, nominative-accusative grammars, serial verb grammars, and active-stative grammars. These strategies are distinct enough to pose serious challenges for linguistic analysis and description. In this approach, the emergence of transitivity is seen as a later evolutionary development, discovering different solutions to the same problem posed by the limitations of the most rudimentary of grammars.

^20^In this respect, Wynn and Coolidge (2004) proposed that working memory may have been enhanced in modern humans, compared to Neanderthals, contributing to the capacity for innovation and experimentation. It is also pointed out by Balari et al. (2013) that enhanced working memory may have enabled recursive syntax. Finally, Benítez-Burraco and Kempe (2018) linked the enhancement of working memory to the emergence of languages with expanded vocabularies and more complex syntax, which are purportedly optimized for conveying complex meanings and know-hows to people not sharing a common ground or a common cultural knowledge.

^21^A reviewer points out that the mainstream view of the evolution of language has shifted away from biological evolution to cultural evolution. While it is true that trends in scientific research often bend in this and then that direction, especially with the questions having to do with nature vs. nurture, our proposal is that both biology and culture are directly involved and that the relevant challenge is to identify phenomena that can be shown to be so intertwined. Our paper presents an attempt in this direction, invoking a complex feedback loop between cultural innovations and biological selection. Importantly, our proposal is detailed enough to allow empirical testing. In this respect, we point out that genetic mutations affecting *FOXP2* and other genes increased synaptic plasticity and neuronal connectivity of the human brain (e.g., Hillert, 2014; Dediu, 2015), particularly in the frontal-striatal network, likely enabling human capacity for more complex language (see also Boeckx and Benítez-Burraco, 2014b). The finding that these networks have a biological foundation, supported by multiple genes, suggests that the emergence of complex syntax/language was not only a cultural invention but also a biological/genetic event.

^22^Gleeson and Kushnick (2018) provided evidence in favor of sexual selection *via* female preference for less aggressive males, leading to reduced sexual dimorphism, but only in the societies where females have relatively high social status, high enough to be able to choose. Furthermore, this effect is more robust where food resources are more secure. In the case of food scarcity, even when females can exert a choice, the tendency is toward selecting stronger (more aggressive) males.

^23^As discussed by Progovac and Locke (2009), even compositions that seem to describe females (*laj-kučka* “bark-bitch,” loud, and obnoxious person; *plači-pička* “cry-cunt,” vulgar version of *crybaby*) are in fact typically used in reference to males.

^24^As pointed out by Freeman et al. (2009) and others, the research on coprophenomena (coprolalia and copropraxia) is very limited and leaves large gaps and many unanswered questions. The same is true of the studies of insult, swearing, and profanity more generally (e.g., Mohr, 2013; Bergen, 2016).
